# Experimental Combined Immunotherapy of Tumours with Major Histocompatibility Complex Class I Downregulation

**DOI:** 10.3390/ijms19113693

**Published:** 2018-11-21

**Authors:** Adrianna Grzelak, Ingrid Polakova, Jana Smahelova, Julie Vackova, Lucie Pekarcikova, Ruth Tachezy, Michal Smahel

**Affiliations:** Department of Genetics and Microbiology, Faculty of Science, Charles University, BIOCEV, 25250 Vestec, Czech Republic; grzelaka@natur.cuni.cz (A.G.); ingrid.polakova@natur.cuni.cz (I.P.); smahelovaj@natur.cuni.cz (J.S.); julie.vackova@natur.cuni.cz (J.V.); lucie.pekarcikova@natur.cuni.cz (L.P.); tachezr@natur.cuni.cz (R.T.)

**Keywords:** MHC-I, cancer immunotherapy, DNA immunization, CpG ODN, α-galactosylceramide, tumor-associated macrophages

## Abstract

Combined immunotherapy constitutes a novel, advanced strategy in cancer treatment. In this study, we investigated immunotherapy in the mouse TC-1/A9 model of human papillomavirus type 16 (HPV16)-associated tumors characterized by major histocompatibility complex class I (MHC-I) downregulation. We found that the induction of a significant anti-tumor response required a combination of DNA vaccination with the administration of an adjuvant, either the synthetic oligodeoxynucleotide ODN1826, carrying immunostimulatory CpG motifs, or α-galactosylceramide (α-GalCer). The most profound anti-tumor effect was achieved when these adjuvants were applied in a mix with a one-week delay relative to DNA immunization. Combined immunotherapy induced tumor infiltration with various subsets of immune cells contributing to tumor regression, of which cluster of differentiation (CD) 8^+^ T cells were the predominant subpopulation. In contrast, the numbers of tumor-associated macrophages (TAMs) were not markedly increased after immunotherapy but in vivo and in vitro results showed that they could be repolarized to an anti-tumor M1 phenotype. A blockade of T cell immunoglobulin and mucin-domain containing-3 (Tim-3) immune checkpoint had a negligible effect on anti-tumor immunity and TAMs repolarization. Our results demonstrate a benefit of combined immunotherapy comprising the activation of both adaptive and innate immunity in the treatment of tumors with reduced MHC-I expression.

## 1. Introduction

With the advent of immunotherapy, new prospects have been opened up for successful cancer treatment. Increasing evidence shows that the efficacy of immunotherapeutic drugs can be enhanced by combined applications, which are believed to support the anti-tumor response at multiple sites [1,2]. Further advances include optimization of immunotherapy timing as well as deeper investigation and better understanding of tumor microenvironment biology [2].

It is well established that cluster of differentiation (CD) 8^+^ cytotoxic T lymphocytes (CTLs) are the main anti-tumor effectors that are able to kill cancer cells in terms of their binding with the major histocompatibility complex class I (MHC-I) molecules presenting antigenic peptides on a tumor-cell surface. As MHC-I downregulation is a mechanism widely used by tumor cells to avoid the recognition by T cells and finally escape the immune surveillance [3], upregulation of MHC-I cell-surface expression has recently been proclaimed the critical step to improve response to cancer immunotherapy [4].

DNA vaccines constitute an attractive immunotherapeutic tool since they are stable, safe and reproducible; they also involve low-cost production and easy modification of an antigen. Although low immunogenicity is the major obstacle that limits large-scale application of DNA immunization [5], the potency of these vaccines can be enhanced by adjuvants, for example, ligands for Toll-like receptors (TLRs) [6]. Synthetic oligodeoxynucleotides (ODNs) carrying unmethylated immunostimulatory CpG motifs and levamisole (LMS) can activate TLR-9 and TLR-2, respectively, which leads to the enhancement of T helper 1 (Th1) immune response and stimulation of antigen presenting cells (APC) [7,8]. Another compound with strong adjuvant activity is α-galactosylceramide (α-GalCer) binding specifically to the CD1d protein present on APCs. These cells subsequently activate invariant natural killer T (iNKT) cells [9]. Through the production of vast amounts of cytokines of both Th1 pro-inflammatory (e.g., interferon γ; IFN-γ, or tumor necrosis factor α; TNF-α) and Th2 anti-inflammatory (e.g., interleukins (IL), such as IL-4, IL-10 and IL-13) responses followed by downstream activation of natural killer (NK) cells, B cells, dendritic cells (DCs) and conventional T cells, iNKT cells bridge the innate and adaptive immunity and affect both MHC-I positive and negative tumor cells, which are the targets for CTL and NK cell lysis, respectively. This is an especially attractive approach for elimination of heterogeneous tumor cells with respect to the MHC-I variability [10].

T cell immunoglobulin and mucin-domain containing-3 (Tim-3) was initially identified as a molecule expressed on the differentiated Th1 cells [11]. The results on preclinical cancer models have revealed that Tim-3 acts as an immune checkpoint receptor whose expression on dysfunctional CD8^+^ T cells and regulatory T lymphocytes (Tregs) attenuates the Th1 response [12]. Moreover, dysregulation of Tim-3 expression is not limited to T cells, as ample evidence shows that it affects functionality of innate immunity cells, including NK cells, DCs, mast cells and macrophages (MΦs) [13], thus making Tim-3 a suitable candidate for cancer immunotherapy [12].

MΦs are the most abundant tumor infiltrating cells that can represent up to 50% of tumor cell mass and their presence at the tumor site is associated with poor prognosis in the major types of solid tumors [14]. Regarding the anti-tumor immunity, classically activated MΦs (M1) are considered advantageous due to their ability to eliminate tumor cells. This type of MΦs produce pro-inflammatory cytokines, such as IL-12 and TNF-α and the free radical NO, a product of inducible nitric oxide synthase (iNOS) [15]. As the tumor progresses, M1-like MΦs may change their functional phenotype to the alternatively activated tumor-promoting M2 profile associated with the increased Tim-3 expression [16]. Furthermore, expression of high levels of arginase 1 is the hallmark of M2 MΦs [15]. This enzyme competes with iNOS for the common substrate-arginine-and directs metabolism of this amino acid to the production of ornithine, a precursor of collagen [17]. Tumor-associated macrophages (TAMs) mostly resemble the M2 phenotype in already established tumors [18] and promote tumor growth and metastasis [19]. Therefore, an increasing attention is paid to the strategies that affect TAMs in order to increase the efficacy of anti-tumor therapies, for example, by inhibition of MΦ infiltration and their survival in the tumor microenvironment or by blocking pro-tumorigenic activity of TAMs [20]. Moreover, repolarization of M2-like TAMs to the tumoricidal M1 phenotype by modulating the tumor microenvironment has recently attracted much interest [19] because MΦs utilize different mechanisms of tumor-cell killing than T cells and can act when CTL-mediated killing is not evoked or tumor cells are resistant to the CTL activity [21,22]. Indeed, it has been demonstrated in various murine tumor models that upon proper stimulation, MΦs can perform tumoristatic activities both in vitro and in vivo [23,24,25,26,27].

Based on these findings, we investigated a combined immunotherapy that stimulated both adaptive immunity (via DNA immunization) and innate immunity (by administration of TLRs agonists or α-GalCer) in the murine TC-1/A9 tumor model characterized by downregulated MHC-I expression. We also aimed to inhibit immunosuppression by blocking the Tim-3 receptor. Combined immunotherapy reduced the growth of tumors resistant to single therapeutics. In this effect, CD8^+^ T cells, NK1.1^+^ cells and MΦs were involved but the contribution of Tim-3 blockade was marginal.

## 2. Results

### 2.1. ODN1826 and α-GalCer Are Effective Vaccine Adjuvants for DNA Immunization against Tumors with Reduced Expression of MHC Class I Molecules

In initial experiments, mice were immunized with the pBSC/PADRE.E7GGG plasmid by a gene gun 3, 6 and 10 days after inoculation of TC-1/A9 cells. Systemic activation of innate immunity with ODN1585, LMS, ODN1826, or α-GalCer as well as inhibition of immunosuppression by antibody against Tim-3 were performed on the same days as DNA immunization. In accordance with the previous study, DNA vaccination against the E7 oncoprotein did not affect the growth of TC-1/A9-induced tumors. Moreover, the efficacy of DNA immunization was not improved after administration of Tim-3 blockade. A significant tumor growth retardation in immunized mice was found when either ODN1826 or α-GalCer were administered (Figure S1). Blockade of Tim-3 resulted in enhanced anti-tumor response only in mice treated with immunization and α-GalCer (*p* < 0.05, 31 days after inoculation of tumor cells). Additionally, in two immunized mice treated with either ODN1826 or α-GalCer, the tumor did not develop or completely regressed.

**Figure 1:**
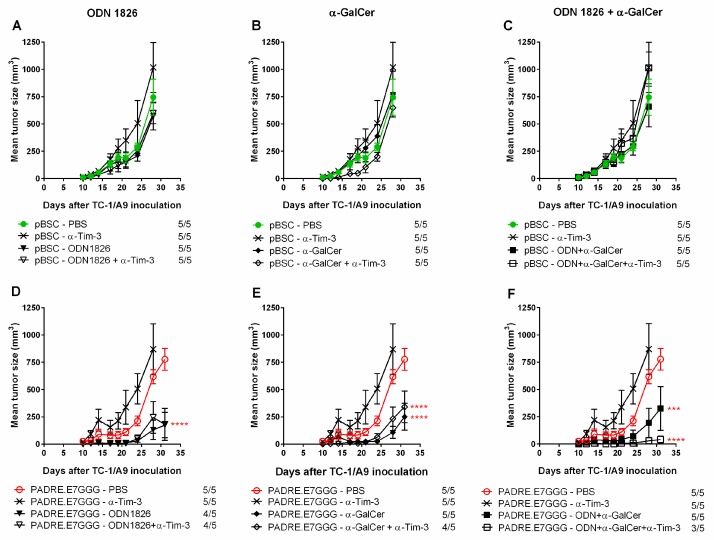
Comparison of the anti-tumor effects induced after the administration of CpG ODN1826 and α-GalCer either alone or in a mix in the non-immunized and immunized mice. Animals (*n* = 5) were injected s.c. with TC-1/A9 cells and immunized 3 times by a gene gun with either the empty pBSC plasmid (referred to as non-immunized mice, **A**–**C**) or pBSC/PADRE.E7GGG (immunized mice, **D**–**F**). Vaccine adjuvants ODN1826 (**A**,**D**), α-GalCer (**B**,**E**), or a mix of ODN1826 and α-GalCer (**C**,**F**) were administered on the same days as DNA vaccines. Some groups received a monoclonal antibody against Tim-3. No. of mice with a tumor/no. of mice in the group is indicated. Bars: ±SEM; *** *p* < 0.001, **** *p* < 0.0001. Statistical significance refers to the comparison with the group immunized with the *PADRE.E7GGG* gene. The experiment was repeated with similar results.

As we demonstrated the significant adjuvant effect only for ODN1826 and α-GalCer, we focused on these two compounds in subsequent experiments. At first, we asked whether these two immunostimulators can exert an anti-tumor response in non-immunized mice (Figure 1A–C). Simultaneously, we evaluated the combination of ODN1826 and α-GalCer (Figure 1C,F). This experiment confirmed the adjuvant efficacy of ODN1826 (Figure 1D) and α-GalCer (Figure 1E) in immunized mice but the combination of these two adjuvants did not further enhance the suppression of tumor growth. Moreover, co-administration of antibody against Tim-3 significantly supported the anti-tumor effect solely in ODN1826 and α-GalCer mixture, resulting in inhibition of tumor growth in 2 out of 5 mice in the group. In non-immunized mice, ODN1826, α-GalCer and anti-Tim-3, neither alone nor in any combination, induced the inhibition of tumor growth.

These data showed that DNA immunization against the E7 oncoprotein was indispensable for combined immunotherapy of tumors with downregulated expression of MHC-I molecules and that combination of two adjuvants, ODN1826 and α-GalCer, did not induce stronger anti-tumor response than single adjuvants.

### 2.2. Delayed Administration of ODN1826 and α-GalCer in Combination Promoted Inhibition of Tumor Growth

**Figure 2:**
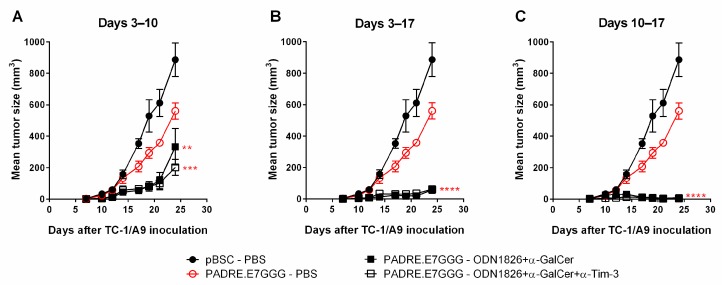
The effects of different dosage and timing protocols. Mice (*n* = 5) were injected with TC-1/A9 cells and immunized by a gene gun. Mice received combinations of ODN1826, α-GalCer and α-Tim-3 3 times on the days of immunization (**A**), 5 times with two additional doses on days 13 and 17 (**B**) and 3 times with a one-week delay following DNA immunization (i.e., on days 10, 13 and 17) (**C**). Bars: ±SEM; ** *p* < 0.01, *** *p* < 0.001, **** *p* < 0.0001. Statistical significance refers to the comparison with the group immunized with the *PADRE.E7GGG* gene. The experiment was repeated with similar results.

**Figure 3:**
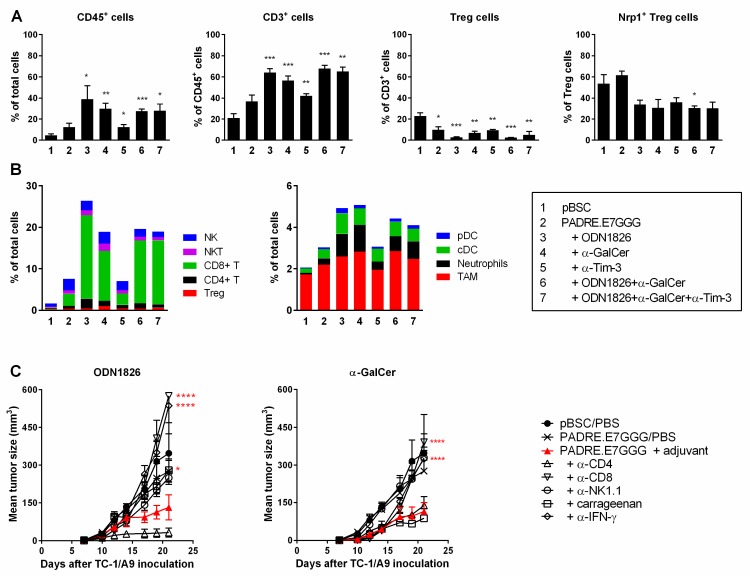
Tumor-infiltrating immune cells and their role in tumor growth. Analysis of tumor-infiltrating cells was performed by flow cytometry (**A**,**B**). Mice (*n* = 4) were injected with tumor cells and immunized by a gene gun. Vaccine adjuvants and anti-Tim-3 were administered on the same days as the DNA vaccines. Tumor cells were isolated on day 12 from non-treated tumors and on days 14–18 from treated tumors and stained with fluorochrome-labeled antibodies. (**A**) Frequencies of CD45^+^ and CD3^+^ cells, Treg (CD4^+^CD25^+^Foxp3^+^) and Nrp1^+^ Treg cells. Statistical significance refers to the comparison with the non-treated (pBSC) group. (**B**) Overview of the mean percentages of the major subpopulations of tumor-infiltrating cells in total cells. (**C**) The effect of in vivo depletion of immune cells and neutralization of IFN-γ on the anti-tumor response induced by immunotherapies with ODN1826 or α-GalCer in the immunized mice (*n* = 5). Vaccine adjuvants were injected on the days of immunization. Statistical significance refers to the comparison with the group that was immunized with the *PADRE.E7GGG* gene and received an adjuvant. Bars: ±SEM; * *p* < 0.05, ** *p* < 0.01, *** *p* < 0.001, **** *p* < 0.0001.

In spite of the substantial efficacy of combined immunotherapy against TC-1/A9 cells, most mice still developed a tumor. Therefore, we also tested modifications in the number and timing of doses. To this end, we compared previously used injection of the ODN1826 plus α-GalCer mixture (supplemented with anti-Tim-3 in some groups) on days of immunization (i.e., 3 doses delivered 3, 6 and 10 days after inoculation of tumor cells, Figure 2A) with injection of 5 doses on days 3, 6, 10, 13 and 17 (Figure 2B) and 3 doses on days 10, 13 and 17 (Figure 2C). Application of two additional doses enhanced the anti-tumor response in comparison to three doses on days of DNA immunization but even higher improvement was achieved with three doses delayed by one week in comparison with the original schedule. After postponing the administration of immunostimulatory compounds, a portion of initially developed tumors partially regressed until day 24 but they subsequently progressed in all mice. Co-administration of anti-Tim-3 did not improve the anti-tumor effect in any group. In summary, the highest efficacy of the adjuvants was achieved when administered one week after DNA immunization.

### 2.3. Immunotherapy Induced Infiltration of Tumors with Various Immune Cells that Differently Affected Tumor Growth

To find cells with anti-tumor activity, we first studied infiltration of tumors with immune cells by flow cytometry using two panels of antibodies identifying the main subpopulations of lymphoid and myeloid cells. We isolated cells from the tumors of the treated mice 14–18 days after inoculation of TC-1/A9 cells, because in this period tumor growth was slowed down and tumors partially regressed in some mice. In the tumors of non-treated mice analyzed 12 days after tumor-cell inoculation, CD45^+^ cells constituted about 5% of total live cells (Figure 3A). After DNA immunization, the numbers of these cells approximately doubled and further increase was recorded when ODN1826 or α-GalCer were administered on days of DNA immunization. A mix of ODN1826 and α-GalCer did not outperform the effect of single adjuvants. CD3^+^ cells constituted about 20% of CD45^+^ cells in non-treated mice, almost 40% in DNA-immunized mice and approximately 60% in mice receiving combined immunotherapy (Figure 3A). Antibody against Tim-3 influenced neither infiltration of CD45^+^ cells nor a proportion of CD3^+^ cells.

In non-treated tumors, the population of CD45^+^ cells comprised particularly TAMs (defined as CD11b^+^F4/80^+^Gr1^−/low^) and NK cells (CD3^−^NK1.1^+^) (Figure 3B). After DNA immunization, all of the detected subpopulations were increased and this increase was enhanced after co-administration of ODN1826 or α-GalCer. CD8^+^ T cells were the predominant subpopulation of infiltrating cells in treated tumors, especially after combined immunotherapy. Of the myeloid cells (CD3^−^), TAMs were the most abundant but the highest increase was found for neutrophils (CD11b^+^F4/80^−^Gr1^high^) and classical DCs (cDC; CD11c^+^F4/80^−^Gr1^−^MHC-II^+^). Increase in plasmacytoid DCs (pDC; CD11c^+^Gr1^−/low^F4/80^−^CD11b^−^CD317^+^) was observed as well.

Intratumoral regulatory T cells (Treg; CD4^+^CD25^+^Foxp3^+^) were also increased after immunotherapy (Figure 3B) but the proportion of Tregs in CD3^+^ cells was reduced from approximately 20% in non-treated tumors up to 3% in tumors treated with DNA immunization and ODN1826 (Figure 3A). As neuropilin 1 (Nrp1) has been identified as a critical factor for the stability of Treg cells and their suppression of anti-tumor immunity [28] we tested Nrp1 expression on Tregs. While in tumors of non-treated and DNA-immunized mice, about 60% of Tregs expressed Nrp1, after combined immunotherapy, Nrp1 expression was decreased to about 40% (Figure 3A).

To further investigate the mechanisms underlying the anti-tumor efficacy of ODN1826 or α-GalCer in the immunized mice that were injected with TC-1/A9 tumor cells, we depleted in vivo CD4^+^, CD8^+^, or NK1.1^+^ cells with monoclonal antibodies and MΦs by administration of carrageenan. Furthermore, we performed IFN-γ neutralization with monoclonal antibody. After administration of ODN1826, depletion of CD8^+^ cells and neutralization of IFN-γ completely abolished the anti-tumor effect of combined immunotherapy and inoculation of carrageenan and anti-NK1.1 partially restored tumor growth. On the contrary, antibody depletion of CD4^+^ cells increased the anti-tumor response. In mice treated with DNA immunization and α-GalCer, antibodies against CD8, NK1.1 and IFN-γ induced tumor growth but carrageenan and anti-CD4 did not have a marked effect (Figure 3C).

In summary, after combined immunotherapy, TC-1/A9-induced tumors were increasingly infiltrated with various immune cells. CD8^+^ cells, NK1.1^+^ cells and IFN-γ contributed to the anti-tumor effect of both ODN1826 and α-GalCer but MΦs reduced tumor growth only after administration of ODN1826 and this adjuvant stimulated also immunosuppressive CD4^+^ cells.

### 2.4. Combined Immunotherapy Did Not Substantially Enhance Either Systemic or Intratumoral Activation of CD8^+^ Cells

**Figure 4:**
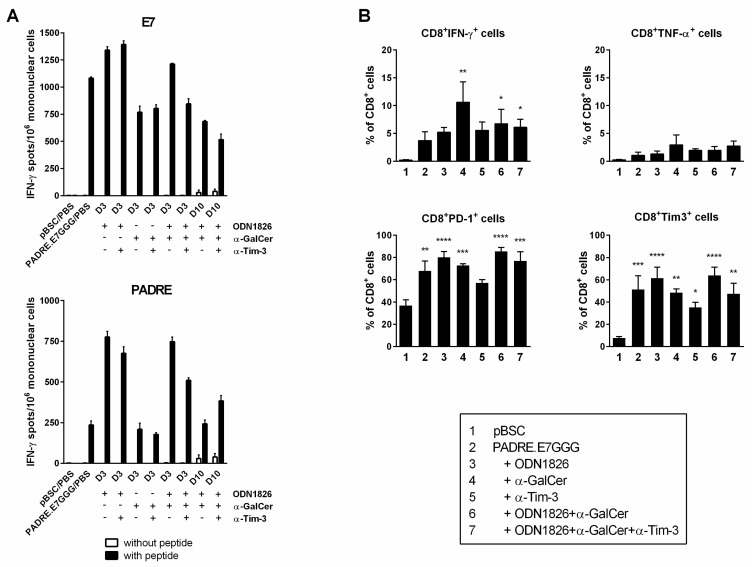
Activation of CD8^+^ T cells by combined immunotherapy and characterization of tumor-infiltrating CD8^+^ T cells. (**A**) Analysis of activated CD8^+^ cells by an ELISPOT assay. Mice (*n* = 3) were immunized by a gene gun on days 3, 6 and 10 and inoculated with ODN1826, α-GalCer and anti-Tim-3 on the days of immunization (D3) or with a one-week delay following DNA immunization (D10). Eight days after the last immunization, mononuclear cells were prepared from pooled splenocytes, restimulated with peptides and IFN-γ-producing-cells were detected. Columns, mean of triplicate samples; bars, ± SEM. The experiment was repeated with similar results. (**B**) Analysis of intratumoral CD8^+^ T cells by flow cytometry. The experiment was performed as in Figure 3. Columns, mean of four samples; bars, ± SEM; * *p* < 0.05, ** *p* < 0.01, *** *p* < 0.001, **** *p* < 0.001. Statistical significance refers to the comparison with the non-treated (pBSC) group.

As CD8^+^ T lymphocytes were the predominant cells that infiltrated tumors after combined immunotherapy, we tested their systemic activation by an ELISPOT assay detecting the IFN-γ production in mononuclear cells isolated from spleens of non-tumor-bearing mice. For this testing, we applied the immunization schedules used in our previous immunotherapeutic experiments. Mononuclear splenocytes were activated with either the E7_49–57_ peptide carrying the immunodominant H-2D^b^-restricted epitope that activates CD8^+^ T cells specific for the human papillomavirus type 16 (HPV16) E7 oncoprotein or the PADRE peptide representing a universal helper epitope activating CD4^+^ T cells (Figure 4A). Immunization of mice with the empty pBSC plasmid served as a negative control and restimulation of mononuclear splenocytes from these mice with either peptide did not result in their activation to produce IFN-γ. A significant induction of both E7-and PADRE-specific reactions was observed when mice received the PADRE.E7GGG DNA vaccine. Administration of ODN1826 on days of DNA immunization augmented the PADRE-specific immunity about three times but this strong activation resulted in only slight stimulation of the response of CD8^+^ T cells. α-GalCer did not promote activation of either CD4^+^ or CD8^+^ T cells (it rather slightly reduced stimulation of the E7-specific response). Activation of CD8^+^ cells induced by DNA immunization was not also enhanced by combined immunotherapy that included antibody against Tim-3 and/or a mix of ODN1826 and α-GalCer or by one-week-delayed administration of adjuvants.

Next, we evaluated the functional state of CD8^+^ T cells in tumors by flow cytometry determining the production of IFN-γ and TNF-α cytokines and the expression of PD-1 and Tim-3 immune checkpoints. While in non-treated mice, the expression of IFN-γ and TNF-α in CD8^+^ T cells was negligible, both cytokines were increased after immunotherapy (reaching at least 3% and 1% positive cells, respectively; Figure 4B) but this increase was relatively low. The mean expression of PD-1 and Tim-3 in non-treated mice was about 35% and 8%, respectively and it was significantly increased up to about 80% and 60% after immunotherapy, respectively (Figure 4B). Tim-3 blockade slightly reduced upregulation of these checkpoints.

These data also show that activation of specific CD8^+^ T cells induced by DNA immunization was not further significantly enhanced by adjuvants. High expression of immune checkpoints on intratumoral CD8^+^ lymphocytes after immunotherapy could be responsible for their relatively low expression of IFN-γ and TNF-α.

### 2.5. Immunotherapy Induced Polarization of TAMs into M1 MΦs

**Figure 5:**
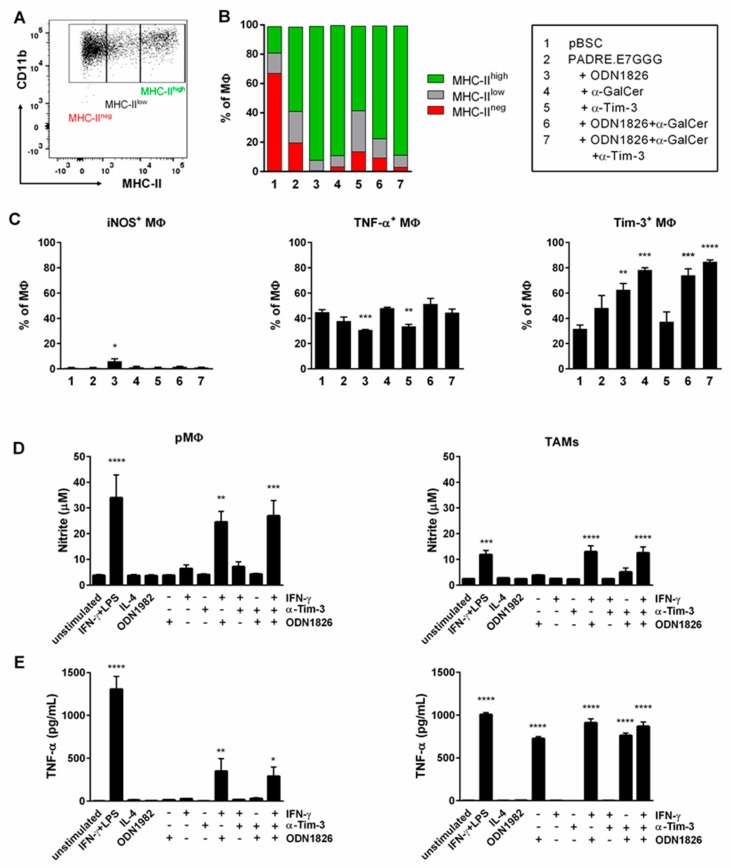
Characterization of TAMs. After immunotherapy, TAMs were isolated from tumors and analyzed by flow cytometry. The experiment was performed as in Figure 3. (**A**) Gating of an MHC-II marker. (**B**) Overview of mean percentages of TAM subpopulations distinguished by MHC-II expression. (**C**) Frequencies of iNOS^+^, TNF-α^+^ and Tim-3^+^ MΦs; columns, mean of four samples. TAMs were also isolated from non-treated tumors and stimulated in vitro. The nitrite (**D**) and TNF-α (**E**) concentrations were measured in the supernatants by Griess reagent and ELISA test, respectively. pMΦs were used for comparison. Columns, mean of 3 independent experiments. Bars, ± SEM; * *p* < 0.05, ** *p* < 0.01, *** *p* < 0.001, **** *p* < 0.0001. Statistical significance refers to the comparison with the non-treated (pBSC)/unstimulated group.

We monitored polarization of TAMs by staining of MHC-II molecules that are used as a marker of M1 MΦs. In flow cytometry analysis of TAMs, we distinguished cells with low and high MHC-II expression (Figure 5A). In tumors of non-treated mice, the proportion of M2 MΦs was more than 60% (Figure 5B). After DNA immunization, we found about 20% of M2 MΦs and their proportion further decreased after the addition of ODN1826 and/or α-GalCer. iNOS and TNF-α are other markers of M1 MΦs [29]. However, iNOS expression was significantly increased only in the group with the strongest M1 polarization, that is, after DNA immunization accompanied by ODN1826 administration (Figure 5C) and TNF-α expression was not enhanced after immunotherapy. TNF-α was produced by about 40–50% of TAMs but in tumors treated with DNA immunization and ODN1826 or anti-Tim-3, its expression was significantly reduced to 30–35% (Figure 5C). Tim-3 blockade did not markedly promote M1 polarization, although the Tim-3 receptor was expressed on about 30% of TAMs in non-treated tumors and this expression was significantly augmented to 60–85% after administration of ODN1826 and/or α-GalCer.

To study the effect of ODN1826 on MΦs, in vitro stimulations of TAMs and peritoneal MΦs (pMΦs) were performed. pMΦs are one of the best-studied MΦ populations. They are characterized by plasticity that allows them to acquire specific phenotypes upon stimulation with different cytokines. The isolated pMΦs and TAMs were stimulated in vitro with ODN1982, ODN1826, IFN-γ and anti-Tim-3 to investigate polarization to the anti-tumor M1 phenotype. We hoped to elucidate the function of Tim-3 in this polarization as Tim-3 was shown to be expressed on the surface of elicited pMΦs from C56BL/6 mice [30]. For analysis of MΦ stimulation, we used enzymatic activity of iNOS and TNF-α production. Incubation with IFN-γ together with lipopolysaccharide (LPS; a positive control for M1 polarization) and with IFN-γ+ODN1826 led to the induction of iNOS expression (Figure 5D) and secretion of TNF-α (Figure 5E) in both TAMs and pMΦs. ODN1826 alone induced production of TNF-α and slight activation of iNOS but only in TAMs. Blockade of Tim-3, stimulation with IL-4 (used as a positive control for M2 polarization), or incubation with the control ODN1982 did not influence activation of any M1 marker.

To summarize, these data show that TAMs were polarized in vivo into M1 MΦs after combined immunotherapy. Both TAMs and pMΦs from naïve mice were polarized to the M1 phenotype upon in vitro stimulation with the combination of ODN1826 and IFN-γ but ODN1826 alone significantly induced only TNF-α production in TAMs. Tim-3 blockade did not influence M1 polarization.

### 2.6. Co-Culture of TAMs with TC-1/A9 Tumor Cells Enhanced iNOS and Arginase Activity

**Figure 6:**
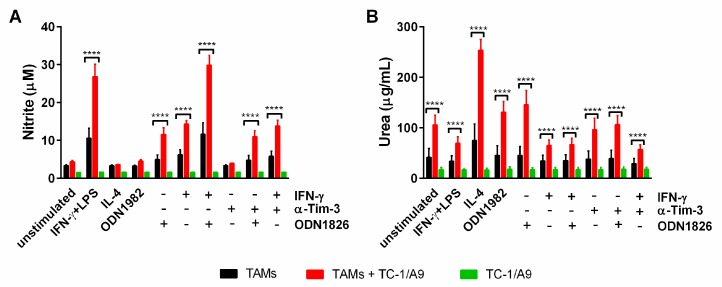
In vitro modulation of iNOS and arginase activity in the co-culture of TAMs with TC-1/A9 cells. Co-cultures as well as control cells, i.e., TAMs and TC-1/A9 cells alone, were stimulated for 44 h. The nitrite concentration was determined by Griess reagent (**A**) and urea was quantified by the microplate method (**B**). Columns, mean of three independent experiments; bars ± SEM; **** *p* < 0.0001. Statistical significance refers to the comparison of co-cultures with TAMs alone.

Expression of iNOS and arginase are hallmarks of M1 and M2 phenotypes, respectively [29]. In fact, little is known about the modulation of activity of both enzymes with respect to the interaction of TAMs with tumor cells. To address this question, we performed co-culture experiments, where TAMs isolated from TC-1/A9-induced tumors were cultured in vitro with TC-1/A9 cells in stimulation variants comprising IFN-γ, a TLR agonist (LPS or ODN1826) and anti-Tim-3, followed by measurement of nitrite and urea concentrations (Figure 6), which are the reaction products of iNOS and arginase, respectively. Stimulation of co-cultures with IFN-γ+LPS and IFN-γ+ODN1826 resulted in further enhancement of nitrite production in comparison to the production of this iNOS metabolite in TAMs alone (Figure 6A). Moreover, co-culture of tumor cells with TAMs stimulated with either IFN-γ or ODN1826 significantly induced iNOS activity, while slight increase of nitrite production was detected in TAMs stimulated alone. Blockade of Tim-3 in the co-cultures, either alone or in combination with ODN1826 or IFN-γ, did not affect iNOS activity. Nitrite concentration in co-cultures upon stimulation with IL-4 or control ODN1982 remained unchanged with respect to TAMs alone. These results indicate that TC-1/A9 cells promoted the iNOS induction in TAMs activated by IFN-γ or ODN1826.

In TAMs isolated from the TC-1/A9-induced tumors, urea production was only augmented after stimulation with IL-4 (Figure 6B, *p* < 0.05), a classical activator of the M2 phenotype. This effect was markedly enhanced by co-culture of TAMs with TC-1/A9 tumor cells (Figure 6B). The co-culture alone significantly enhanced urea production in TAMs and after stimulation of co-cultures with ODN1826, further enhancement was recorded. IFN-γ inhibited the stimulatory activity of co-culture. Blockade of Tim-3 did not affect the arginase activity in co-cultures. The urea background in TC-1/A9 cells was lower than in TAMs and was not influenced by any stimulation. In summary, co-culture alone stimulated arginase in TAMs. While IL-4 promoted this effect, IFN-γ reduced arginase activity.

### 2.7. Enhancement of IFN-γ Expression in Tumors Correlated with Induction of Ido1 Expression

**Figure 7:**
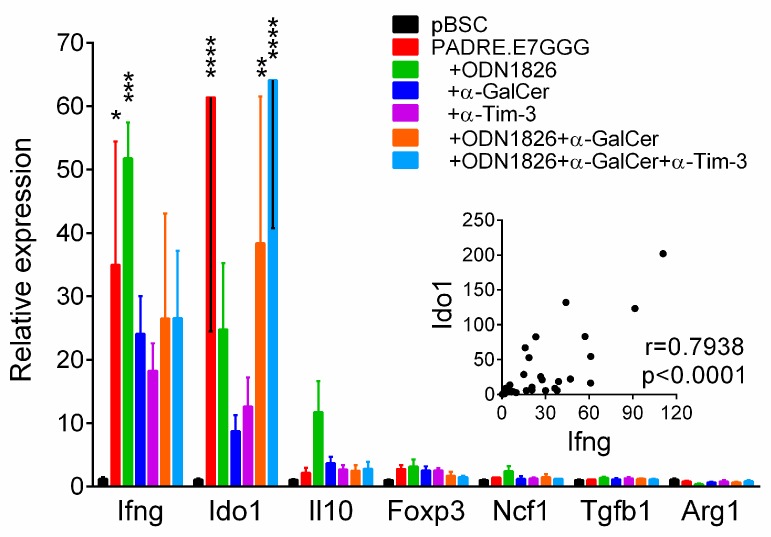
Analysis of gene expression in tumors by RT-qPCR. The experiment was performed as in Figure 3. Columns, mean of 4–6 samples; bars, ± SEM; * *p* < 0.05, ** *p* < 0.01, *** *p* < 0.001, **** *p* < 0.0001. Relative expression and statistical significance refer to the comparisons with the non-treated (pBSC) group. The inserted graph shows the correlation between *Ifng* and *Ido1* expression.

Next, we analyzed immune reactions in the tumor microenvironment by RT-qPCR. As we induced only temporary inhibition of tumor growth by combined immunotherapy, we supposed that immunosuppressive mechanisms outperformed antitumor immunity during tumor development. Therefore, we focused this analysis on the expression of some genes that could be associated with immunosuppression, namely *Tgfb1* and *Il10* producing cytokines TGF-β1 and IL-10, respectively, *Foxp3* that is activated in Treg cells, *Ido1* and *Arg1* encoding enzymes indoleamine 2,3-dioxygenase 1 (IDO1) and arginase 1, respectively and *Ncf1* coding for the p47(phox) subunit of the inducible NADPH oxidase type 2 (NOX2) produced in myeloid derived suppressor cells (MDSC) and Treg cells. The expression of *Ifng* encoding IFN-γ was also tested. The highest induction of expression after immunotherapy was found for *Ido1* and *Ifng* (Figure 7). Despite high variability, the expression of these two genes significantly correlated (Figure 7). The expression was also increased for *Il10* and *Foxp3* but when compared with the pBSC-treated group by multiple comparisons, no significant difference was observed. These data suggest that IFN-γ induced by immunotherapy initiated not only activation of anti-tumor reactions but also immunosuppression that was mediated particularly by IDO1 in the tumor microenvironment.

## 3. Discussion

It is increasingly evident that highly efficient cancer immunotherapy must include activation of both adaptive and innate immunity accompanied by the inhibition of immunosuppressive mechanisms [31,32,33]. In this study, combined immunotherapy was designed to target tumors induced by subcutaneous (s.c.) injection of mouse oncogenic TC-1/A9 cells, characterized by reversible downregulation of MHC-I molecules. Since, in our previous studies, we had demonstrated that TC-1/A9- induced tumors were poorly sensitive to DNA vaccination against the E7 oncoprotein [34] and in tumors developed from parental TC-1 tumor cells, the effect of DNA immunization was markedly enhanced by systemic delivery of ODN1826 or LMS [35], we tested these immunostimulatory drugs against MHC-I-deficient TC-1/A9 cells in this study. Moreover, as we identified a substantial proportion of NK and NKT cells in immune cells infiltrating TC-1-induced tumors [36], we also evaluated the effect of ODN1585 used for the activation of NK cells and α-GalCer inducing NKT cells.

After the initial screening of four immunostimulatory drugs combined with DNA immunization, we performed our next experiments with the two most efficient compounds, ODN1826 and α-GalCer. As their primary target cells are different—TLR-9-expressing cells, mainly MΦs, DCs and NK cells for ODN1826 and iNKT cells for α-GalCer—we hoped for their possible synergistic or additive effect. We found that both ODN1826 and α-GalCer had to be combined with DNA immunization for the induction of anti-tumor effect. Reinis et al. demonstrated the anti-tumor effect of monotherapy with immunostimulatory drugs against TC-1/A9 cells but they applied ODN1826 or ODN1585 intratumorally [37] or administered β-GalCer intraperitoneally (i.p.) in different dosage and timing protocols [38]. A mix of ODN1826 and α-GalCer did not promote DNA immunization more efficiently than single adjuvants. Only when the number of adjuvant doses was increased from three to five or when three adjuvant doses were administered with a one-week delay after DNA immunization, tumor growth was further inhibited but this reduction was transient. The delayed delivery of adjuvants probably enables activation of adaptive immunity induced by DNA vaccination that subsequently cooperates with innate immunity stimulated by adjuvants [38]. This enhancement is similar to the impact of delayed PD-1/PD-L1 blockade [36,39].

Given that most cells infiltrating TC-1 tumors are M2-polarized MΦs [40] that produce the Tim-3 receptor (our unpublished result), we included the Tim-3 blockade into our combined immunotherapy in order to support the activity of adjuvants in M1 polarization of TAMs. As this polarization into the anti-tumorigenic phenotype could substantially contribute to the anti-tumor effect irrespective of MHC-I expression on tumor cells, this study focused on repolarization of TAMs and their involvement in anti-tumor immunity in this study. However, we found only weak and inconsistent effect of anti-Tim-3 treatment on anti-tumor response. Moreover, flow cytometric and functional in vitro analysis of TAMs did not show any benefit of Tim-3 blockade for M1 polarization and anti-tumor stimulation of TAMs.

In an attempt to detect cells and mechanisms contributing to temporary tumor regression, we analyzed the tumor microenvironment during this period. In comparison with non-treated mice, all detected populations of lymphoid and myeloid cells were increased after DNA immunization. Following the addition of ODN1826 and/or α-GalCer, CD8^+^ T cells in particular were further increased. When we tested the systemic activation of E7-specific CD8^+^ T cells by DNA immunization, we found a weak enhancement induced by ODN1826 but α-GalCer rather reduced this response, which corresponded to results obtained after the combination of α-GalCer administration with DNA vaccination against *Trypanosoma cruzi* trans-sialidase surface antigen [41]. Repeated administration of α-GalCer could be responsible for the impairment of immunity induced by a DNA vaccine [42].

In vivo depletion and neutralization showed an involvement of CD8^+^ T cells, NK1.1^+^ cells and IFN-γ in anti-tumor immunity induced by both ODN1826 and α-GalCer. However, TAMs were only necessary for the anti-tumor effect elicited by ODN1826 stimulation. Which cells directly killed tumor cells is doubtful. Despite MHC-I downregulation in oncogenic TC-1/A9 cells, these cells could be a target for CD8^+^ T cells because MHC-I expression has been upregulated in vivo [34]. However, Bercovici and Trautmann challenged the idea that the main function of CD8^+^ T cells in tumor regression is direct tumor cell killing [43]. Instead, they proposed a role of MΦs and neutrophils in this process and supported their suggestion experimentally in the TC-1 tumor model [27]. In that study, they showed a dynamic cooperation between CD8^+^ T cells and myeloid cells in the microenvironment of regressing tumors and demonstrated killing of tumor cells by TAMs via TNF-α production and phagocytosis. Similar results showing cooperation between CD8^+^ T cells and TAMs and a critical role of TAMs in elimination of TC-1-induced tumors were also observed in another study after vaccination with peptides [44].

Further flow cytometric analysis of CD8^+^ T cells from regressing tumors showed increased production of IFN-γ and TNF-α after immunotherapy but only in a small portion of cells and no difference was found between DNA immunization and combined immunotherapy. Increased PD-1 expression suggests that most cells were activated by immunotherapy but as the majority of these cells also expressed Tim-3, they were probably exhausted [45]. Additionally, other immunosuppression mechanisms may have outweighed the induced anti-tumor reactions, such as PD-L1 and IDO1 expression (found by RT-qPCR analysis) that may have been induced by IFN-γ elicited by immunotherapy.

In parallel to the in vivo experiments, we tested the in vitro activation of MΦs by ODN1826, anti-Tim-3 and IFN-γ that were supposed to mimic the in vivo immunotherapies. Besides TAMs isolated from TC-1/A9-induced tumors, pMΦs were examined in these experiments as the reference cells. We observed that neither IFN-γ nor ODN1826 could significantly induce iNOS in both types of MΦs but their combination resulted in high NO production. This result corresponds to the concept of ‘priming’ by IFN-γ and ‘triggering’ by various TLR agonists, indicating that MΦ activation requires two signals for inducing iNOS activity [46,47].

TNF-α is a pro-inflammatory cytokine, which can negatively regulate the expression of M2 MΦ genes, both in vivo and in vitro [48]. In contrast to the high amounts of TNF-α produced by TAMs and pMΦs upon stimulation with IFN-γ plus the TLR ligand, ODN1826 differently affected these MΦ populations in our in vitro experiments. We observed low amounts of TNF-α secreted by ODN1826-activated pMΦs, while ODN1826-activated TAMs produced bulk of this cytokine. This result suggests that different mechanisms govern the production of TNF-α in both types of MΦs, which may be ascribed to the functional heterogeneity of these cells or presence of mechanisms that neutralize TNF-α in a long-term culture of ODN1826-activated pMΦs [49].

Despite the growing evidence of NO contribution to MΦ-mediated tumor cell killing [24,25], the role of this molecule in cancer remains controversial, as its pro- or anti-tumor activities depend strongly on concentration [50]. To delineate how tumor cells can affect NO production by MΦs, we set up co-culture experiments of TAMs with TC-1/A9 cells. Additionally, we tested arginase activity as this enzyme competes with iNOS for arginine but performs antagonistic activity [17] or may work as the iNOS modulator to overcome the exacerbating NO production [51,52]. In culture with tumor cells, TAMs produced significantly more NO than in mono-culture, provided that they were simultaneously challenged with IFN-γ plus TLR agonists. Tumor cells also induced iNOS activity in IFN-γ- or ODN1826-stimulated TAMs. However, whether such activated TAMs are tumoricidal remains questionable. As previously reported, tumor cells can provide a co-stimulatory signal for increased NO production by activated pMΦs but this NO enhancement did not correlate with cytotoxicity exerted on tumor cells in vitro [53]. Furthermore, while in in vivo immunotherapies against TC-1-induced tumors, NO did not affect the tumor regression [27], NO produced by in vitro activated pMΦs can act as one of the cytotoxic mediators against tumor cells [24,25]. On the other hand, tumor cells may also suppress NO production by pMΦs [22].

In our study, arginase activity in TAMs seemed to be upregulated more easily than iNOS. While NO production in co-culture with TC-1/A9 cells was limited to the simultaneous stimulation with IFN-γ, the TLR ligand, or their combination, arginase activity in TAMs was upregulated by co-culture with tumor cells regardless of further stimulation. We hypothesize that such arginase upregulation may be either due to more efficient arginine utilization by M2 MΦs [17] or influenced by the presence of other cell types, which were detected in the adherent population of CD45^+^ cells. A recent study has described a CD11b^+^Gr1^+^F4/80^−^ cell subpopulation that adheres strongly to the plastic surface but is unable to differentiate into TAMs and additionally resembles more MDSCs than MΦs [54]. Although the authors do not report on whether this cell subpopulation expresses either of the enzymes studied in our experiments, it is well established that MDSCs can upregulate arginase but also iNOS [55].

MΦs are gaining increasing interest as they are numerous in tumors and possess potential for adaptation to perform anti-tumor activities. In this study, we showed that TAMs isolated from TC-1/A9-induced tumors are plastic cells that can be polarized to the M1 phenotype by combined immunotherapy that is efficient against tumor cells with reduced MHC-I expression. Thus, TAMs should not be neglected when designing the immunotherapy against tumors with downregulated MHC-I molecules.

## 4. Materials and Methods

### 4.1. Preparation of Reagents and Media

The TLR-9 agonists ODN1826 (class B; TCCATGACGTTCCTGACGTT) and ODN1585 (class A; GGGGTCAACGTTGAGGGGG) carrying immunostimulatory CpG motifs and a negative control, ODN1982 (TCCAGGACTTCTCTCAGGTT) without CpG motifs, were synthetized with a phosphorothioate-modified backbone (Generi Biotech, Hradec Kralove, Czech Republic) and dissolved in phosphate-buffered saline (PBS; ODN1826 and ODN1982) or deionized water (ODN1585). α-GalCer (Abcam, Cambridge, UK, ab144262) was solubilized in dimethyl sulfoxide (DMSO; Sigma-Aldrich, St. Louis, MO, USA) by heating at 80 °C for 20 min and sonication in an ultrasonic bath until complete dissolution.

TC-1/A9 cells were grown in high glucose Dulbecco’s Modified Eagle’s Medium (DMEM; Sigma-Aldrich, St. Louis, MO, USA, D6429) supplemented with 10% fetal bovine serum (FBS; Biosera, Nuaille, France, FB-1090) and 100 U/mL penicillin and 100 μg/mL streptomycin (DMEM-K). MΦs were maintained in DMEM F12 medium (Biosera, Nuaille, France LM-D1222) with 10% FBS and antibiotics as mentioned above (DMEM F12/10).

### 4.2. Mice

Seven- to eight-week-old female C57BL/6NCrl mice (Charles River, Sulzfeld, Germany) were used in the immunization experiments. Animals were maintained under standard conditions and in accordance with the guidelines for the proper treatment of laboratory animals at the Animal Facility of the Czech Center of Phenogenomics (BIOCEV, Vestec, Czech Republic). All animal experimental procedures were carried out in compliance with Directive 2010/63/EU and animal protocols were approved by the Sectoral Expert Committee of the Czech Academy of Sciences for Approval of Projects of Experiments on Animals (reference number 46/2016, 16th May 2016).

### 4.3. Tumor Cell Line

Tumor development in mice was induced with the TC-1/A9 clone [34] that was derived from mouse TC-1 cell line. TC-1 cells were generated by transformation of primary C57BL/6 mouse lung cells with the HPV16 *E6/E7* oncogenes and activated *H-ras* [56]. From a TC-1-induced tumor that developed in a mouse preimmunized against the E7 antigen, the TC-1/A9 clone was selected based on a reduced surface expression of MHC-I molecules. This MHC-I downregulation is reversible and can be restored with the IFN-γ treatment [34].

### 4.4. Plasmids

The pBSC [57] and pBSC/PADRE.E7GGG [58] plasmids were used for immunization of mice. The *PADRE.E7GGG* fusion gene consists of the mutated HPV16 *E7* gene (*E7GGG*) containing three-point mutations resulting in substitutions D21G, C24G and E26G in the Rb-binding site of the E7 oncoprotein [57] and the sequence encoding the Pan DR helper epitope (PADRE) designed in silico [59]. The plasmids were transformed into competent *E. coli* XL-1 blue strain, cultured in Luria Broth Medium with 100 μg/mL of ampicillin and purified with the NucleoBond Xtra Maxi Kit (Macherey-Nagel, Duren, Germany, 740414).

### 4.5. Combined Immunotherapy Experiments

C57BL/6NCrl mice (five per group) were challenged with 3 × 10^4^ TC-1/A9 tumor cells suspended in 0.15 mL PBS by s.c. injection into the back under anesthesia (day 0). Mice were immunized with the pBSC/PADRE.E7GGG plasmid by a gene gun (Bio-Rad, Hercules, CA, USA) on days 3, 6 and 10 after tumor-cell inoculation. DNA vaccination was applied at a discharge pressure of 400 psi into the shaven skin of the abdomen. Each immunization consisted of two shots delivering together 2 μg of plasmid DNA. The empty pBSC plasmid was used as a negative control.

The immunized mice were injected i.p. with four different vaccine adjuvants dissolved in 200 µl PBS. ODN1826 and ODN1585 were administered at a dose of 50 μg, LMS (Sigma-Aldrich, St. Louis, MO, USA, L9756) at a dose of 20 μg and α-GalCer at a dose of 2 μg. Some groups of mice were injected i.p. with 200 μg/200 µl PBS of anti-Tim-3 monoclonal antibody (clone RMT 3-23; Bio X Cell, West Lebanon, NH, USA). Control mice received PBS. The immunostimulatory compounds ODN1826 and α-GalCer were also administered in a mix. Different schedules and numbers of doses in combined immunotherapy were tested as specified for each experiment.

In the depletion experiments, the following doses of monoclonal antibodies (Exbio, Prague, Czech Republic) were injected i.p.: 100 μL of anti-CD4 (clone GK1.5), 100 μL of anti-CD8 (clone 2.43) and 400 μL of anti-NK1.1 (clone PK136). To deplete MΦs, 1 mg of carrageenan IV (Sigma-Aldrich, St. Louis, MO, USA, 22049) dissolved in 200 µl PBS was inoculated i.p. [60]. Neutralization of IFN-γ was achieved with 300 μg of monoclonal antibody per mouse (clone P4-6A2, Bio X Cell, West Lebanon, NH, USA). Depletions and neutralizations were performed on days 4, 7, 11, 14, 18 and 21 after tumor-cell inoculation.

Tumor growth was monitored three times a week and tumor volume was calculated using the formula (π/6) (*a* × *b* × *c*), where a, b, c are length, width and height of the tumor.

### 4.6. Isolation of TAMs

For in vitro experiments on TAMs, C57BL/6NCrl mice were injected s.c. with 3 × 10^5^ TC-1/A9 cells. Non-necrotic tumors were excised as soon as they reached 10 mm in diameter (i.e., on days 13–14). To obtain single-cell suspension, removed tumors were washed in PBS, cut into <3 mm pieces and disaggregated with 1 mg/mL collagenase NB 8 (SERVA, Heidelberg, Germany, 17456) and 100 μg/mL DNase I (SERVA, Heidelberg, Germany, 18535) in RPMI-1640 medium (without FBS) at 37 °C using a gentleMACS Octo Dissociator (Miltenyi Biotec, Bergisch Gladbach, Germany). The obtained cell suspension was filtered through a 70-μm mesh (Miltenyi Biotec, Bergisch Gladbach, Germany). Red blood cells were lysed with the ACK buffer (0.15 M NH^4^Cl, 10 mM KHCO^3^, 0.5 M EDTA, pH 7.2–7.4), which was followed by positive selection of CD45^+^ cells using anti-CD45 antibody-conjugated magnetic beads (Miltenyi Biotec, Bergisch Gladbach, Germany, 130-097-153) and an autoMACS Pro Separator device (Miltenyi Biotec, Bergisch Gladbach, Germany) according to the manufacturer’s instruction. The selected CD45^+^ cells were plated on a 10-cm dish at a concentration of 1.5 × 10^6^ cells/mL adjusted with the complete DMEM F12/10 medium and allowed to adhere to the plastic at 37 °C and 5% CO_2_ for 3–4 h. Then, the non-adherent cells were removed by extensive washing with warm PBS. The adherent cells were collected by gentle scraping in cold 10 mM EDTA/PBS and 5 × 10^5^ cells per well were plated into a 24-well plate in complete DMEM F12/10 medium. The cells were incubated at 37 °C and 5% CO_2_ overnight for the subsequent in vitro experiments.

### 4.7. Isolation of pMΦs

Peritoneal cells were collected by peritoneal cavity lavage. C57BL/6NCrl mice were injected i.p. with 1 mL of 3% Brewer thioglycolate broth (Sigma-Aldrich, St. Louis, MO, USA, 70157) 96 h prior collection. pMΦs were enriched by adhesion to plastic for 3 h by plating 5 × 10^5^ cells per well into a 24-well plate containing 750 μL of DMEM F12/10. Non-adherent cells were removed by washing with warm PBS and adherent cells were incubated in a fresh medium at 37 °C and 5% CO_2_ overnight.

### 4.8. In Vitro Stimulations of TAMs and pMΦs

For in vitro stimulations, cells were cultured in a final volume of 1 mL of DMEM-K in a 24-well plate. Different variants of cell activation were tested with the following concentrations of reagents: 5 μg/mL of ODNs, 10 μg/mL of LMS, 10 μg/mL of anti-Tim-3, 10 ng/mL of LPS (Sigma-Aldrich, St. Louis, MO, USA, L4391) and 200 U/mL IFN-γ (PeproTech, Rocky Hill, NJ, USA, 315-05). Stimulation with LPS+IFN-γ served as a positive control for M1 MΦs and activation of MΦs to M2 type was achieved by incubation with 25 ng/mL IL-4 (PeproTech, Rosky Hill, NJ, USA, 214-14). Unstimulated cells served as a negative control. pMΦs were stimulated for 72 h and TAMs for 48 h. Supernatants were collected, centrifuged for 5 min at 350× *g* and used in aliquots for NO and TNF-α assays.

### 4.9. NO Measurement

NO was determined by measurement of its stable metabolite NO_2_^−^ in supernatants by Griess reagent composed of 0.2% naphthylethylenediamine dihydrochloride (Sigma-Aldrich, St. Louis, MO, USA, 222488) and 2% sulphanilamide (Sigma-Aldrich, St. Louis, MO, USA, S9251) in 5% phosphoric acid. Equal volumes of the supernatant from in vitro cell stimulations and Griess reagent were mixed and incubated in the dark at room temperature (RT) for 10 min. The absorbance was measured at 540 nm using a microplate reader (Tecan, Männedorf, Switzerland). The nitrite concentration was determined by using the standard curve of sodium nitrite (0–100 μM; Sigma-Aldrich, St. Louis, MO, USA, 31443).

### 4.10. TNF-α Enzyme-Linked Immunosorbent Assay (ELISA)

TNF-α concentration was determined in the supernatants from in vitro cell stimulations using sandwich ELISA (eBioscience, San Diego, CA, USA, 88-7324-22) according to the manufacturer’s protocol.

### 4.11. IFN-γ Enzyme-Linked Immunospot (ELISPOT) Assay

An IFN-γ ELISPOT assay was performed on mononuclear cells isolated from pools of splenocytes (3 mice per group) according to the protocol described previously [36]. Cells were incubated with either 0.1 μg/mL of the E7_49–57_ peptide (RAHYNIVTF, > 96% pure; Clonestar Biotech, Brno, Czech Republic) derived from the HPV16 E7 oncoprotein or 1 μg/mL of the PADRE peptide (AKFVAAWTLKAAA, >81% pure; GenScript, Piscataway, NJ, USA) at 37 °C in 5% CO2 for 24 h.

### 4.12. Co-Culture of TC-1/A9 Tumor Cells with TAMs

TAMs from TC-1/A9-induced tumors were isolated according to the protocol decribed above. The concentration of the collected adherent cells was adjusted to 1 × 10^6^ cells/mL with DMEM F12/10 and 100 μL of this cell suspension was distributed into the 96-well plate and incubated at 37 °C in 5% CO_2_ for the subsequent co-culture experiments. On the next day, the adherent cells were washed with warm PBS and some of them were overlaid with 1 × 10^4^ TC-1/A9 tumor cells in 100 μL of DMEM-K. Control cells, that is, TAMs without tumor cells and TC-1/A9 cells alone, were incubated in DMEM-K as well. After the adhesion of TC-1/A9 cells, the cells (i.e., TC-1/A9, MΦs and their co-culture) were washed with PBS and subsequently 100 μL of stimulatory compounds with the concentrations described above were applied for the next 44 h. Afterwards, the supernatants were collected for the measurement of nitrite by the Griess reagent and the cells were washed with PBS for the arginase microplate assay. Incubation of DMEM-K with stimulatory compounds without cells followed by measurement with the Griess reagent was performed in order to eliminate the interference with this reagent.

### 4.13. Arginase Microplate Assay

Arginase activity was measured in terms of urea quantification using the microplate method [61] on 1 × 10^5^ of TAMs, 1 × 10^4^ of TC-1/A9 cells, or their co-culture after 44-h stimulation. The absorbance was measured at 540 nm using a microplate reader and the urea concentration was calculated using the calibration curve ranging from 0 to 320 μg/mL.

### 4.14. Flow Cytometry

The preparation of a single cell suspension from a tumor tissue was performed as described previously [36] (see also isolation of TAMs). Four panels of fluorescent-labeled antibodies were used to identify the main cell populations infiltrating the tumor (Table 1). Staining was performed in 96-well plates. Prior to surface staining, the cells were stained for viability with Fixable Viability dye eFluor 455UV (eBioscience, San Diego, CA, USA, 65-0868-14) in PBS. Staining of surface markers was followed by a fixation and permeabilization step if needed. To detect the nuclear Foxp3 transcription factor, the cells were treated with the Fixation/Permeabilization Concentrate (eBioscience, San Diego, CA, USA, 00-5123-43) diluted 1:3 with the Fixation/Permeabilization Diluent (eBioscience, San Diego, CA, USA, 00-5223-56). Cytokine and iNOS production were observed after incubation with the IC Fixation buffer (eBioscience, San Diego, CA, USA, 00-8222-49). In both cases, a washing step with the Permeabilization buffer (eBioscience, San Diego, CA, USA, 00-8333-56) followed the fixation and permeabilization. Subsequently, the cells were measured on an LSRFortessa (BD Biosciences, San Diego, CA, USA) flow cytometer and the results were analyzed with FlowJo v10.4.2 software (BD Biosciences, San Diego, CA, USA).

### 4.15. Quantification of mRNA Expression by RT-qPCR

Specific primers for the detection of target genes (Table 2) were designed and evaluated with SYBR green chemistry by the Gene Core-qPCR and dPCR Core Facility (BIOCEV, Vestec, Czech Republic), which also performed all qPCR reactions. As reference genes for normalization, *Tbp* (TATAA-box binding protein), *Ywhas* (Tyrosine 3-tryptophan 5-monooxygenase activation protein) and *Rplp* (Ribosomal protein P0, large) were selected from the Reference Gene Panel (Mouse) (TATAA Biocenter, Goteborg, Sweden, A102). For the selection of these genes with the most stable expression, 36 tumors induced by TC-1 or TC-/A9 cells were utilized. To ensure variability of the tumor microenvironment with respect to immunotherapy, some mice were treated with DNA immunization, LMS, ODN1826, ODN1585, carrageenan, or antibody against IFN-γ as described above. After the excision of tumors from mice, a representative part of samples was immediately placed into the RNAlater Solution (Thermo Fisher Scientific, Waltham, MA, USA, AM7021) and stored at 4 °C for a maximum of 14 days prior to RNA isolation. Then, samples were disrupted by a rotor-stator homogenizer (Omni TH, Kennesaw, GA, USA) in a lysis buffer from the NucleoSpin RNA kit (Macherey-Nagel, Duren, Germany, 740955) and total RNA was isolated. One microgram of RNA was reverse transcribed in a 20-µL reaction using the TATAA GrandScript cDNA Synthesis kit (TATAA Biocenter, Goteborg, Sweden, A103b) according to the manufacturer’s instructions. The amplifications of cDNA of the target genes and three reference genes were done simultaneously in duplicates in a 384-well microplate format of the LightCycler 480 Real-Time PCR System (Roche Diagnostics, Basel, Switzerland). Ten microliters of the reaction solution contained 1× TATAA SYBR Grandmaster mix (TATAA Biocenter, Goteborg, Sweden), 400 nM of each of primer and 2 µL cDNA (diluted 10×). The standard program was used (95 °C for 30 s followed by 40 cycles of 95 °C for 5 s, 60 °C for 15 s, 72 °C for 10 s and melting curve). For the control of the genomic DNA background, the ValidPrime assay (TATAA Biocenter, Goteborg, Sweden) was done. The data processing and relative quantification of mRNA expression were performed using the GenEx v6 software (TATAA Biocenter, Goteborg, Sweden).

### 4.16. Statistical Analysis

Tumor growth was analyzed by two-way analysis of variance (ANOVA) and Sidak multiple comparisons. Intergroup comparisons from flow cytometry and in vitro stimulations of peritoneal cells and CD45^+^ adherent cells were made by one-way ANOVA and Dunnett multiple comparisons. Co-culture and RT-qPCR experiments were analyzed by two-way ANOVA and Dunnett multiple comparisons. The Spearman coefficient was calculated for the analysis of the correlation between *Ifng* and *Ido1* expression. Results were considered significantly different if *p* < 0.05. Calculations were performed using the GraphPad Prism 6 software (GraphPad Software, San Diego, CA, USA).

## 5. Conclusions

This study demonstrates that for immunotherapy of tumors with MHC-I downregulation, the combined activation of both adaptive and innate immunity is needed. Despite negligible sensitivity of these tumor cells to DNA immunization, CD8^+^ T cells activated by immunization played an important role in the anti-tumor response elicited by combined immunotherapy. In addition, NK1.1^+^ cells and MΦs repolarized to the M1 phenotype were involved in the inhibition of tumor growth. However, Tim-3 blockade did not significantly contribute to the anti-tumor effect. As tumor growth was reduced only transiently, further efforts should enhance the combined immunotherapy to handle immunosuppression that probably prevailed in progressing tumors.

## Figures and Tables

**Table 1:** List of antibodies used for flow cytometry.

Antigen	Conjugate	Clone	Source	Staining	Panels			
CD11b	BV421	M1/70	BioLegend, 101251	Surface		●		●
CD11c	BV650	N418	BioLegend, 117339	Surface		●		
CD25	APC	PC61.5	eBiosciences, 17-0251-81	Surface	●			
CD3	APC-Cy7	145-2C11	BioLegend, 100330	Surface	●	●	●	●
CD317	APC	927	BioLegend, 127015	Surface				
CD4	BV510	RM4-5	BioLegend, 100559	Surface	●		●	
CD45	Alexa Fluor 700	30-F11	BioLegend, 103128	Surface	●	●	●	●
CD8	FITC	53-6.7	BD Biosciences, 553031	Surface	●	●	●	
F4/80	BV510	BM8	BioLegend, 123135	Surface		●		
Foxp3	PE	FJK-16s	eBiosciences, 12-5773-82	Nuclear	●			
Gr-1	PE/BV786 ♦	RB6-8C5	BioLegend, 108407/BD Biosciences, 740850 ♦	Surface		●		♦
IFN-γ	BV421	XMG1.2	BD Biosciences, 563376	Intracellular			●	
MHC-II	PerCP-Cy5.5/PE-Cy7 ♦	M5/114.15.2	BioLegend, 107625/BioLegend 107629 ♦	Surface		●		♦
NK1.1	BV650	PK136	BioLegend, 108736	Surface	●		●	
iNOS	Alexa Fluor 488	CXNFT	eBiosciences, 53-5920-80	Intracellular				●
Nrp1	BV421	3E12	BioLegend, 145209	Surface	●			
PD-1	PE-Cy7	29F.1A12	BioLegend, 135215	Surface			●	
Tim-3	APC	RMT3-23	BioLegend, 119706	Surface			●	●
TNF-α	PE-DAZZLE594	MP6-XT22	BioLegend, 506346	Intracellular			●	●

● ♦, antibody present in a panel.

**Table 2:** List of target genes and primer sequences for qPCR assays.

Target Gene	Reference Sequence (NCBI ID)	Forward Primer 5′ → 3′	Reverse Primer 5′ → 3′	Amplicon (bp)
*Ifng*	NM_008337.4	TTCCTCATGGCTGTTTCTGG	CACCATCCTTTTGCCAGTTC	148
*Ido1*	NM_001293690.1	GTCTGGAGAAAGCCAAGGAA	ATATGCGGAGAACGTGGAAA	81
*Il10*	NM_010548.2	GGTGAGAAGCTGAAGACCC	ATGGCCTTGTAGACACCTTG	137
*Foxp3*	NM_001199347.1	ACCTGGCTGGGAAGATG	TCCCGAGGAGCAGACC	124
*Ncf1*	NM_010876.4	TGTTCCTGGTTAAGTGGCAG	GGTGTGGGATGACTCTGTTC	144
*Tgfb1*	NM_011577.2	TCAGACATTCGGGAAGCAG	AAGGTAACGCCAGGAATTGT	135
*Arg1*	NM_007482.3	ATGGAAGAGTCAGTGTGGTG	GGGAGTGTTGATGTCAGTGT	128

NCBI, National Center for Biotechnology Information; *Ifng*, interferon gamma; *Ido1*, indoleamine 2,3-dioxygenase 1; *Il10*, interleukin 10; *Foxp3*, forkhead box P3; *Ncf1*, neutrophil cytosolic factor 1; *Tgfb1*, transforming growth factor, beta 1; *Arg1*, arginase, liver.

## Supplementary Materials

Supplementary materials can be found at http://www.mdpi.com/1422-0067/19/11/3693/s1.
