# Supplementary material for: Experimental Combined Immunotherapy of Tumours with Major Histocompatibility Complex Class I Downregulation
Source: Int J Mol Sci. 2018 Nov 21;19(11):3693. doi: 10.3390/ijms19113693 (PMC6274939; doi:10.3390/ijms19113693)

Figure S1. The anti-tumor effect of single vaccine adjuvants and Tim-3 blockade in DNA immunized mice. Mice (n=5) were injected s.c. with TC-1/A9 cells (day 0) and immunized 3 times by a gene gun with 2 μg of pBSC/PADRE.E7GGG on days 3, 6, and 10. The empty plasmid pBSC was used as a negative control (**A**). Monoclonal antibody against Tim-3 was administered alone (**A**) or together with vaccine adjuvants – ODN1585 (**B**), LMS (**C**), ODN1826 (**D**), or α-GalCer (**E**) – delivered on the days of immunization. No. of mice with a tumor/no. of mice in the group is indicated. Bars: ±SEM; * p < 0.05; ** p < 0.01; ***p < 0.001. Statistical significance refers to the comparison with the group immunized with the PADRE.E7GGG gene. The experiment was repeated with similar results.

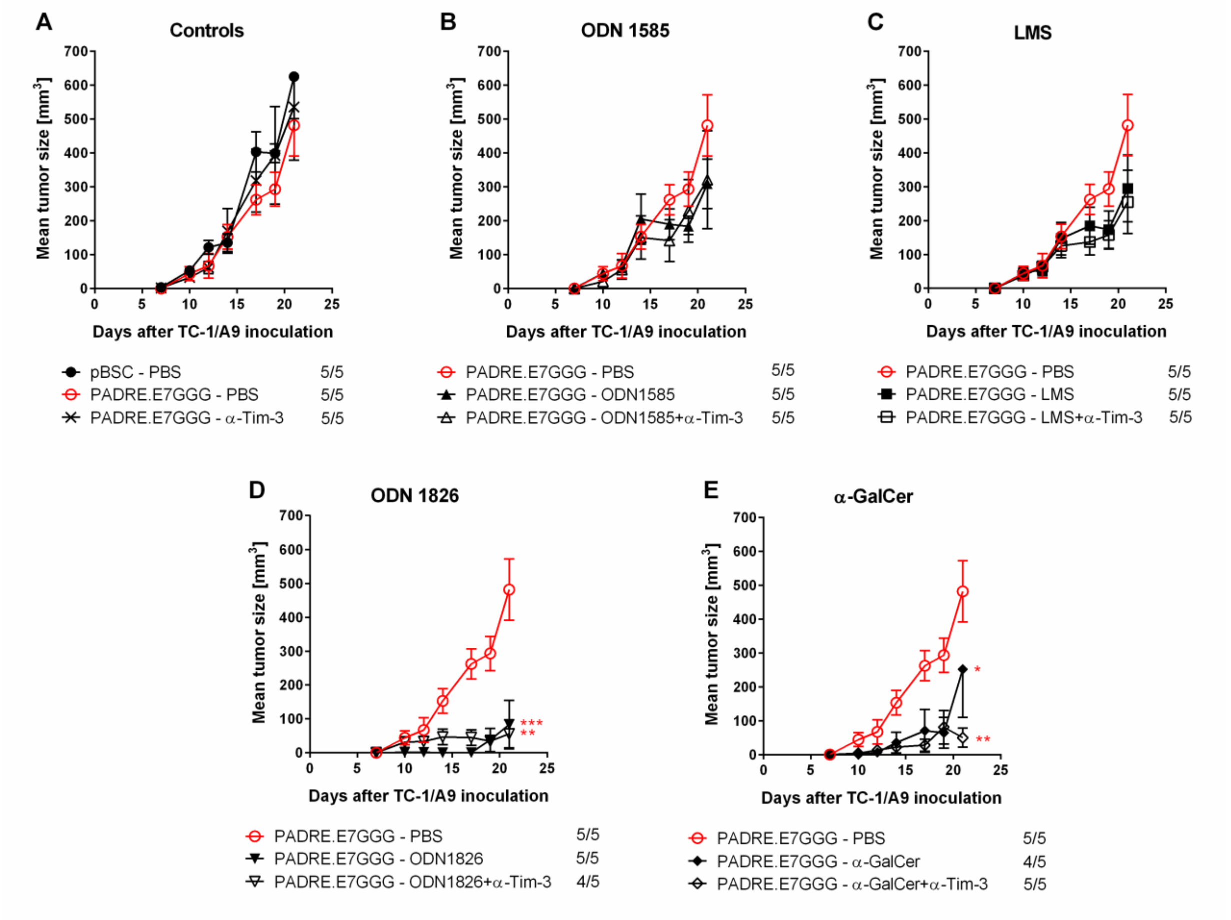

Supplement: Supplementary file 1 [file ijms-19-03693-s001.pdf]
